# Supplementary material for: Evaluating the Image Quality of Neck Structures Scanned on Chest CT with Low-Concentration-Iodine Contrast Media
Source: Tomography. 2022 Nov 28;8(6):2854–63. doi: 10.3390/tomography8060239 (PMC9785131; doi:10.3390/tomography8060239)
Supplement: Supplementary file 1 [file tomography-08-00239-s001.zip › tomography-1965058-supplementary.pdf]

Supplemental Material

Table S1: In machine A, comparing the group with 240 iodine contrast media (CM) and the group with 320 iodine CM.

	Group with 240 iodine CM in machine A (n=77)	Group with 320 iodine CM in machine A (n=107)	*p*-value
Sharpness	2.92 ± 0.24	2.96 ± 0.18	0.28
Noise	2.34 ± 0.46	2.32 ± 0.41	0.76
Overall image quality	4.26 ± 0.59	4.28 ± 0.47	0.84

CM = contrast media

Table S2: In machine A, comparing the group with 240 iodine CM and relatively low kVp (90≥) and the group with 320 CM media and relatively high kVp (100≤).

	Group with 240 iodine CM and relatively low kVp (90≥) in machine A (n=34)	Group with 320 iodine CM and relatively high kVp (100≤) in machine A (n=65)	*p*-value
Sharpness	2.93 ± 0.22	2.98 ± 0.10	0.14
Noise	2.32 ± 0.41	2.30 ± 0.42	0.79
Overall image quality	4.25 ± 0.51	4.28 ± 0.44	0.73

Table S3: In machine B, comparing the group with 240 iodine CM and the group with 320 iodine CM.

	Group with 240 iodine CM in machine B (n=82)	Group with 320 iodine CM in machine B (n=100)	*p*-value
Sharpness	2.90 ± 0.24	2.96 ± 0.18	0.10
Noise	2.19 ± 0.32	2.20 ± 0.33	0.82
Overall image quality	4.09 ± 0.47	4.16 ± 0.38	0.32

Table S4: In machine B, comparing the group with 240 iodine CM and relatively low kVp (90≥) and the group with 320 iodine CM and relatively high kVp (100≤).

	Group with 240 iodine CM and relatively low kVp (90≥) in machine B (n=17)	Group with 320 iodine CM and relatively high kVp (100≤) in machine B (n=78)	*p*-value
Sharpness	2.94 ± 0.17	2.94 ± 0.20	0.98
Noise	2.24 ± 0.31	2.18 ± 0.27	0.79
Overall image quality	4.18 ± 0.39	4.12 ± 0.35	0.73

Table S5: Effective doses between Group A and Group B.

	Group A (n=159)	Group B (n=207)	*p*-value
Effective dose	3.38 ± 1.14	3.38 ± 1.44	0.997
